# Preclinical Experiential Global Health Leads to Transformative Learning and Long‑term Impact

**DOI:** 10.5334/aogh.4637

**Published:** 2025-04-28

**Authors:** Marissa Vander Missen, Destiny Resner, Micaela Gaviola, Debra Litzelman, Julia Songok, Jenny Baenziger

**Affiliations:** 1 Indiana University School of Medicine, Indiana, USA; 2 Indiana University Center for Global Health Equity, Indiana, USA; 3 Regenstrief Institute, Inc, Indiana, USA; 4 Moi University School of Medicine, Eldoret, Kenya

**Keywords:** global health, preclinical, early immersive learning, transformative learning theory, global health education, global health ethics

## Abstract

*Background:* The Slemenda Scholars (SS) program at Indiana University School of Medicine offers preclinical students early exposure to global health through a summer program in collaboration with the Academic Model Providing Access to Healthcare (AMPATH). AMPATH Kenya is a 30‑year partnership between a consortium of US and European universities and Moi University in Kenya that provides sustainable, high‑quality care through medical education, clinical care, research, innovation, and community service. The positive impact of electives during medical students’ clinical years is well documented, but the impact of quality, immersive learning in preclinical years has not previously been studied.

*Methods:* A cross‑sectional survey was administered to past participants of the SS program via e‑mails in 2023. Written narratives about the SS program by participants from 1998 to 2023 were evaluated using qualitative analytic methods. Narratives were obtained via internet search and compiled from open‑ended survey responses. Themes were generated on the basis of a constant comparative method using grounded theory and finalized through an iterative consensus process.

*Findings:* Surveys were distributed to 66 SS alumni. In total, 54 responses were received (81.8% response rate). After excluding incomplete responses, 45 were included in the final analyses (68.2%). Respondents indicated they learned more about themselves (mean 4.9; 5 = strongly agree), global health (mean 5), and medicine (4.9) through the SS program and developed skills, including cultural humility and personal resilience. A qualitative review of 50 narratives identified two major themes: impactful experiences and transformative learning.

*Conclusions:* Preclinical global health experiential learning opportunities are impactful and transformative. Immersive learning expands trainees’ perspectives, promotes the development of relational skills with diverse colleagues, and fosters adaptability. Early, immersive global health exposure within the context of established institutional partnerships affirmed or informed a career addressing health disparities both locally and globally.

## Background

U.S. medical students’ interest in global health has been growing for decades. This interest has spurred medical schools to create global health partnerships, add short‑term experiences in global health (STEGH), and increase educational opportunities such as global health “tracks” or electives with a focus on global health [1–4]. Medical students in other countries desire increasing engagement with global health as well [5], and similar interest has been observed in allied health profession trainees [6]. For this study, global health is defined as an interdisciplinary field of study and practice that focuses on improving health for all, regardless of location or ethnicity, consistent with the Alma Ata declaration [7]. An STEGH is defined as a short‑term (usually 2–12 weeks) experience for healthcare trainees or professionals that includes cross‑cultural interaction and exposure to different healthcare systems, projects, or care delivery; STEGHs are often international but may be domestic. The content of STEGHs varies on the basis of the level of training and the degree to which the program is embedded in an established institutional partnership. For residents and fellows, research and clinical electives involving direct or observational patient care have long existed [8–12]. For US medical students in their last year of clinical training, many programs similarly offer clinical or research electives [8, 13, 14]. STEGHs for preclinical medical students are less well described; some are purely volunteer experiences with varying levels of connection to clinical medicine.

STEGHs in the clinical years have been shown to be enriching for personal and professional development [9–11]. STEGHs increase trainees’ self‑evaluated cultural competency and knowledge of social determinants of health and health systems [12, 14]. Numerous studies emphasize the improvement of physical exam, clinical, and diagnostic skills as tangible outcomes from STEGH [9–11].

Evidence suggests that STEGHs shape career choices. Data show participants are more likely to care for patients with public insurance and immigrant populations, as well as work in underserved areas [8–10, 14, 15]. They are also more likely to pursue global or public health, global health advocacy, and public policy [8], with up to 46% of resident participants continuing in global health careers [12]. Participants are more likely to switch from subspecialty to general or primary care practice [8–10] or initially choose primary care [16].

Transformative learning theory describes how STEGHs can reshape a medical trainee’s worldview, career path, and self‑perception. Transformative learning theory characterizes how learners transform existing views through self‑reflection, exposure to new perspectives, and dialogue with those holding differing views [17]. Most data on STEGH outcomes come from participant self‑reflection and surveys [9–11, 13, 18]. Trainees recount meaningful relationships with host country colleagues that fostered dialogue and shifted their perspective on identity, shared humanity, and responsibility for global health equity.

Many programs offer STEGHs for medical students who are in their clinical years or for residents and fellows, but preclinical exposure to high‑quality global health partnerships is less studied. Evidence of STEGHs’ impact on career and interpersonal skills suggests that earlier integration into medical school could increase the benefits [9–15]. For example, preclinical STEGHs might enable students to adjust research and service interests to prepare for global health careers. However, rising demand has led some institutions to create unsustainable short‑term global health experiences, such as drop‑in or “parachute” trips, including “alternative” break trips, which often lack ethical partnership. It is crucial to introduce global health (GH) involvement in connection with ethical practice, especially for preclinical students.

When taking into account increased access to GH education programs, published frameworks for ethical GH education should be considered. Recurrent recommendations include partner‑led development and partnership, prioritizing the self‑identified needs of the host community, provision of program support to alleviate any undue burden on the host, robust predeparture orientation, post‑return reflection, humility of visitors, and bidirectional partnership [19–22]. As an example, the Working Group on Ethics Guidelines for Global Health Training (WEIGHT) guidelines emerged as one such framework for assessing potential benefits and harms related to global health experiences and outlined goals for sending and host institutions, trainees, and sponsors, respectively [23].

This study aimed to assess personal and professional outcomes of an ethically structured STEGH for preclinical medical students, made possible through an established multinational and multi‑institution partnership.

## Methods

### About AMPATH and the slemenda scholars program

The Academic Model Providing Access to Healthcare (AMPATH) is a partnership between a consortium of North American and European universities and Moi University in Kenya with over 30 years’ history in global health development [24, 25]. AMPATH’s long‑term commitment with faculty from member institutions serving multi‑year commitments in‑country, bilateral exchanging of trainees and faculty, and championing local experts and host‑led initiatives align with best practice guidelines for sending institutions.

The Slemenda Scholars (SS) program at Indiana University (IU) School of Medicine provides select preclinical medical students a fully funded opportunity for global health exposure through AMPATH. Started in 1998, the program honors Dr. Charles Slemenda, an IU epidemiologist committed to global health and reducing health disparities. Selection is competitive, involving a written application and interview. Scholars spend 8–10 weeks in Kenya between their first and second years of medical school, engaging in clinical shadowing, program development, didactics, case‑based discussions, education with clinical students concurrently on clerkships, and diverse global health initiatives [24]. They join Kenyan‑led teams for bidirectional learning and build long‑term relationships with guidance from IU faculty based in Kenya. Educational scaffolding is robust; these scholars complete cultural training before travel, are encouraged to take Swahili lessons, have constant support in Kenya, and have structured debriefings upon return. Philanthropy through AMPATH and IU funds the program. The SS program follows an experiential, constructivist learning model, which understands learners’ individualized learning experiences that involve action, emotion, and reflection through individual and group processing, as well as varying identities and social factors that influence their learning outcomes [26].

### Study design

This mixed‑methods study sought to analyze qualitative and quantitative data on the Slemenda Scholars program. This study was submitted to and approved by the corresponding author’s Ethics Institutional Review Board as an exempt project.

### Quantitative analysis

The quantitative data were collected through a cross‑sectional digital survey administered to past participants of the SS program. The survey was distributed via e‑mail, with an initial e‑mail and a reminder in January and February 2023, and a final reminder in November 2023 to increase the response rate prior to final analyses. The survey included questions about demographics, previous global health experiences, and personal and professional outcomes related to the SS program. The survey included one optional open‑ended question: “Please provide any other feedback about your Slemenda Scholars experience. Feel free to include best parts, worst parts, or any other thoughts.”

### Analytic methods

Descriptive statistics were performed on demographic data and categorical question responses. Means and standard deviations were calculated for each of the Likert‑style survey questions, with “1” being “strongly disagree” and “5” being “strongly agree.”

### Qualitative analysis

The qualitative analysis involved reading publicly available narratives about the SS program, including published essays and blog posts, as well as open‑ended survey responses obtained during this study. Writing narrative reflections was recommended but not required for scholars completing the program. Using the constant comparative method, all qualitative data were jointly coded and analyzed, enabling iterative theme generation [27]. Researchers met to discuss and finalize themes, with consensus from ≥ 2 researchers required for inclusion. All articles were read and coded twice, with three meetings to refine consensus themes. Descriptive statistics determined the relative prevalence of each theme in the narratives and responses.

## Findings

### Quantitative analysis

Surveys were emailed to 72 of the 77 alumni of the SS program as of 2023. Three SS alumni (MG, DR, and MV) are the authors of this study and were excluded. Two SS alumni were excluded owing to their having no contact information available. Of the 72 contacted, 6 e‑mails failed to deliver owing to inactive addresses. Fifty‑four responses were received (81.8% response rate; 54/66 successful contacts). After excluding responses that were more than half incomplete, 45 were included in the final analyses (68.1%). Twenty‑five respondents provided narrative responses to the survey’s open‑ended question. See Table 1 for an overview of survey demographics and select data.

**Table 1:** Demographics and descriptive statistics on select data.

DEMOGRAPHICS AND DESCRIPTIVE STATISTICS					
Age (years)	Median age	Average age	Min age	Max age	
	36	36	23	54	
Sex	Woman	Man	Other		
	*N* = 29; 66%	*N* = 15; 34%	0		
GH experience prior to SS?	Yes	No			
	*N* = 22; 49%	*N* = 23; 51%			
Length of prior GH experience	1–2 weeks	3–12 weeks	4–12+ months		
	*N* = 9; 43%	*N* = 9; 43%	*N* = 6; 29%		
Type(s) of prior GH experience	Education	Clinical/healthcare	Community project	GH research	Other
	*N* = 6; 29%	*N* = 12; 57%	*N* = 12; 57%	*N* = 6; 29%	*N* = 1; 5%
Continuation in GH career?	Yes	No	In med school		
	*N* = 30; 70%	*N* = 6; 14%	*N* = 7; 16%		

The median respondent age was 35.5 years (range 23–54), and the majority of respondents were female (66%; 29/44). Although the average age of a Slemenda Scholar varies on the basis of their career path prior to medical school, most are in their mid‑to‑late twenties during the program. Over 80% of respondents were above the age of 30 years, with 38% being over the age of 40 years – suggesting decades of time had elapsed since their participation in the program for most survey respondents. Nearly one‑half reported they had GH experience prior to the SS experience; given where the Slemenda Scholars opportunity fits into the medical school timeline, almost all prior experiences were before matriculation into medical school. A quarter of these prior GH experiences were longer than 12 weeks, and most were clinical or community‑based.

Respondents used a Likert scale from strongly disagree (1) to strongly agree (5) to indicate agreement with statements. Higher scores equated to a more positive experience. One respondent was excluded from this analysis owing to a suspected entry error. The excluded respondent answered “strongly disagree” to all questions, despite writing a positive review about their Slemenda experience in the open‑ended response. As seen in Table 2, respondents indicated that they enjoyed participating in the program and learned more about themselves, global health, and the field of medicine.

**Table 2:** Perceptions of the SS program.

RANGE: 1 (STRONGLY DISAGREE)–5 (STRONGLY AGREE)	
STATEMENT	MEAN (SD)
I enjoyed participating in the Slemenda Scholars program.	4.9 (0.3)
I learned more about myself by participating in the Slemenda Scholars program.	4.9 (0.3)
I learned more about global health by participating in the Slemenda Scholars program.	5 (0.0)
I learned more about the field of medicine by participating in the Slemenda Scholars program.	4.9 (0.3)
Participating in the Slemenda Scholars program changed the trajectory of my career.	4.0 (1.0)

SD, standard deviation.

**Figure 1:**
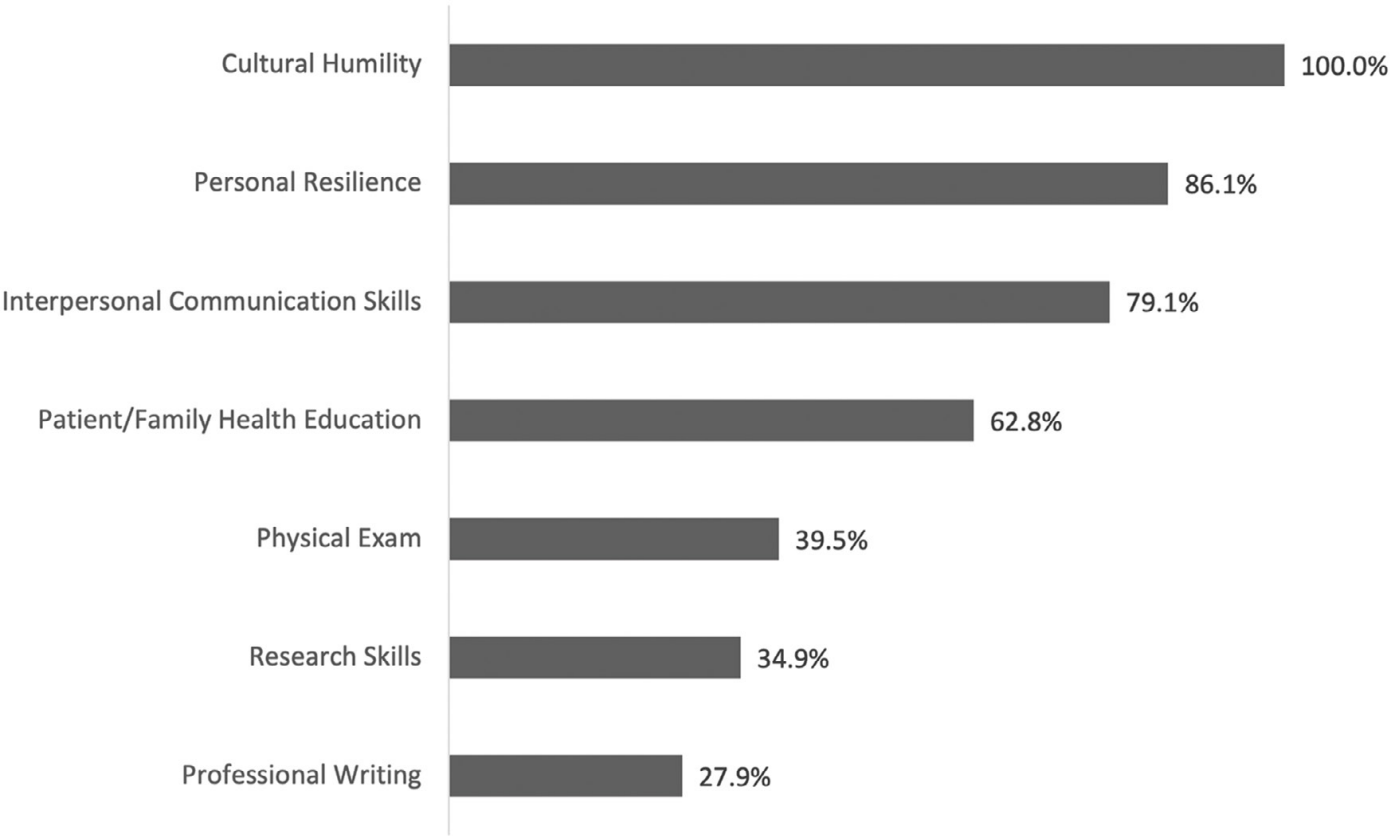
Self‑reported competencies.

The Slemenda Scholars program was also beneficial for knowledge dissemination. Nearly half of respondents reported conducting oral presentations related to their SS projects after the program (60%), and many presented posters (47%). Respondents self‑reported improvements in cultural humility (100%), personal resilience (86%), interpersonal connection (79.1%), patient/family health education (62.8%), physical exam skills (39.5%), research skills (34.9%), and professional writing skills (27.9%) (Figure 1).

Survey respondents reported a high level of continuation in a career in global health (70%). The survey did not have a specific prompt to describe what GH career paths physicians chose. However, in the open‑ended response, some respondents elaborated with the following quotes, demonstrating diverse career involvement:

- “*working during the Ebola epidemic in 2014*”;
- “*been back to Moi University to help in Anesthesia*”;
- *“[my Kenyan] research mentor ended up shaping my career, leading to additional fellowships*”;
- “*I was able to host a student when the Kenyans came to the US*.”

For the respondents who have not continued careers in global health, the most common barriers reported were clinical/professional obligations (67%), travel and professional costs for GH (50%), family obligations (50%), time (33%), and other (33%) – which included coronavirus disease 2019 (COVID‑19) and lack of institutional support. Of the physicians currently working in GH, all respondents indicated that the Slemenda Scholars program had a moderate (34%) or strong (66%) influence on their decision to pursue a career in GH.

### Qualitative analysis

**Figure 2:**
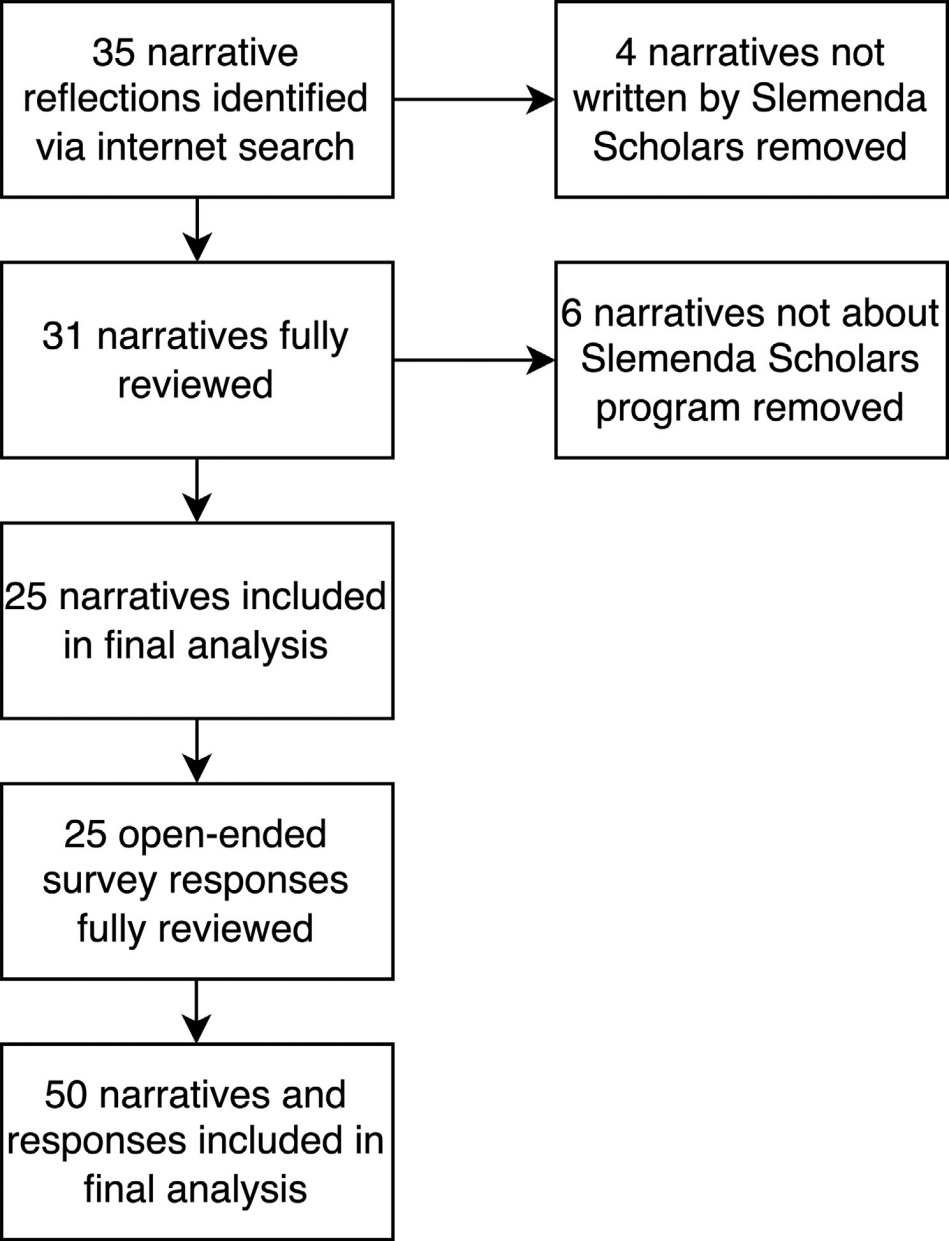
Workflow for identifying narratives about the Slemenda Scholars experience.

A total of 35 publicly available narratives were reviewed. Ultimately, 25 narratives were included in final analyses, after exclusion for content not relevant to Slemenda Scholars program or not written by a Slemenda Scholar. All 25 open‑ended survey responses were also included in the final quantitative analysis (Figure 2).

On the basis of qualitative analyses, two major themes emerged. *Impactful experiences* captured scholars’ accounts of the curricular components of their SS experiences. The four subthemes within *impactful experiences* include experiential learning, didactic learning, professional development, and cross‑cultural interactions. *Transformative learning* included reflections on intangible themes or values instilled through the SS Program. The five subthemes within *transformative learning* are expanding perspectives, motivation for a service‑oriented career, interpersonal skills, innovation, and adaptability.

### Impactful experiences

See Table 3 for full definitions of each subtheme within *impactful experiences*. Of note, the incidence of each theme is stratified by personal narrative versus open‑ended survey response owing to the differences in data. The narratives were written directly after the SS program, with emphasis on detailed reflection as part of the debriefing, whereas the survey responses were collected 1–30 years after the completion of the program and may suffer from recall bias or dilution of detail. This difference in detail is reflected in different proportions of themes.

**Table 3:** Impactful experiences themes.

IMPACTFUL EXPERIENCES	DEFINITIONS	COUNT IN PERSONAL NARRATIVES, *N* (%) *N* = 25 (100%)	COUNT IN SURVEY OPEN‑ENDED RESPONSES, *N* (%) *N* = 25 (100%)	TOTAL *N* = 50 (%)
***Experiential learning***	Informal education occurring in …	21 (84)	6 (24)	27 (54)
Research	Scholarly projects or research endeavors	14 (56)	1 (4)	15 (30)
Clinical experience	Wards or other clinical settings	11 (44)	4 (16)	15 (30)
Community outreach	Volunteer work or site visits outside the hospital	12 (48)	2 (8)	14 (28)
Cultural exploration	Kenya beyond the AMPATH or Slemenda program	7 (28)	0 (0)	7 (14)
***Didactic learning***	Formal instruction on …	16 (64)	6 (24)	22 (44)
AMPATH	Infrastructure and/or history of AMPATH and its programs	14 (56)	5 (20)	19 (38)
Global health education	Global health or Kenyan healthcare system (fireside chats, public health talks, personal or clinical experiences)	5 (20)	4 (16)	9 (18)
***Professional development***		14 (56)	7 (28)	21 (42)
Network‑building	Mentions work with professionals outside of medicine and/or with mentors in AMPATH	14 (56)	4 (16)	18 (36)
Career exploration	Reflects on an experience that changed/affirmed their interest in a field of medicine	3 (12)	3 (12)	6 (12)
***Cross‑cultural interactions***	Collaboration with Kenyans in a …	17 (68)	3 (12)	20 (40)
Professional	Work setting	13 (52)	3 (12)	16 (32)
Personal	Cultural or extraprofessional setting	13 (52)	3 (12)	16 (32)

The most prevalent subtheme in *impactful experiences* was experiential learning. Respondents mentioned clinical experiences and community outreach as methods of experiential learning. Cross‑cultural interactions, both professional and personal; didactic learning; and professional development were nearly evenly represented. Professional development captured SS reflections on their career exploration while in Kenya and building their professional network through interactions with AMPATH and Moi University mentors. Didactic learning included descriptions of seminars about global health or the Kenyan healthcare system, as well as formal education on AMPATH.

Notably, cross‑cultural interactions were written about in rich detail and with more prevalence in the longer personal narratives than in the survey responses, which may be attributed to the educational slant of the preceding questions in the survey influencing the content of survey responses.

Collectively, the *impactful experiences* themes demonstrate that, despite the variability over decades of the SS Program, key components of the curriculum connect the experiences of former participants: formal instruction on the Kenyan healthcare system and global health, collaborative work and leisure with Kenyan colleagues, and exposure to clinical and community‑based AMPATH projects.

### Transformative learning

Participants’ introspection on the abstract takeaways from the SS program produced multiple subthemes within *transformative learning*. See Table 4 for all subthemes with definitions.

**Table 4:** Transformative learning themes.

TRANSFORMATIVE LEARNING	DEFINITIONS	COUNT IN PERSONAL NARRATIVES, *N* (%) *N* = 25 (100%)	COUNT IN SURVEY OPEN‑ENDED RESPONSES, *N* (%) *N* = 25 (%) (100%)	TOTAL *N* = 50 (%)
***Expanding perspectives***	Reflections on experiences in Kenya that strengthened or changed views regarding …	21 (84)	7 (28)	28 (56)
Ethical global health	Importance or understanding of sustainable GH	13 (52)	4 (16)	17 (34)
Impactful patient experiences	Patients	12 (48)	1 (4)	13 (26)
Cultural humility	Kenyan people, culture, and medical system	8 (32)	4 (16)	12 (24)
Health equity	Health disparities or inequities	6 (24)	1 (4)	7 (14)
***Motivation for service‑oriented career***	Expresses future orientation with …	18 (72)	9 (26)	27 (54)
Global health	Desire to continue working in GH	12 (48)	8 (32)	20 (40)
Optimism	Hope for improvement of care and/or quality in GH work	10 (40)	0 (0)	10 (20)
Underserved	Desire to work with underserved communities	3 (12)	1 (4)	4 (8)
***Interpersonal skills***	Reflections on significant/effective interactions in …	7 (28)	5 (20)	12 (24)
Relationship‑building	Social settings	5 (20)	4 (16)	9 (18)
Collaboration	Professional partnerships	4 (16)	2 (8)	6 (12)
***Innovation***	Observations on innovation in Kenyan healthcare, goals for reciprocally implementing Kenyan methods back at home, and ideas for the future of GH	9 (36)	0 (0)	9 (18)
***Adaptability***	Resilience and creativity in a resource‑ or language‑limited setting	4 (16)	1 (4)	5 (10)

The most prevalent subtheme was expanding perspectives, represented by more than half of scholars who reflected on an experience that strengthened or changed their world view. Types of perspective or paradigm shifts identified included ethical global health, health equity, impactful patient experiences, and cultural humility. Narratives demonstrated prevalent motivation for pursuing a service‑oriented career in medicine, whether in global health or other underserved communities. One respondent wrote:

*I feel even more motivated and invigorated to work harder so that one day I can become a bigger part of global efforts like AMPATH and alleviate the suffering that so many individuals endure because they don’t have a choice*.

The theme of interpersonal skills‑building highlighted how SS built relationships and collaborated with their Kenyan counterparts. Less prominent but still notable themes were adaptability and innovation. Examples of adaptability include the opportunities SS had to find creative solutions or build resilience in a resource‑ or language‑limited setting, such as one student working with a local butcher to obtain meat as practice materials for a surgery workshop. The innovation theme underlined how scholars saw opportunities to learn from the Kenyan healthcare system and proposed ideas to reciprocally implement or build upon them, for example:

*I will take the lessons I learned…in this setting and work to apply them to improving healthcare in underserved areas in our own community as well*.

While each scholar’s experiences were diverse, spanning time and changes to the SS program, the transformative learning themes that emerged were stable over time. The bilateral exchange of ideas, culture, and relationships in the SS program continues to shift trainees’ worldviews, ignite passion for health equity and global health, and build self‑efficacy for their future roles in the field. Table 5 contains illustrative quotes of the themes in *transformative learning.*

**Table 5:** Illustrative quotes from transformative learning themes.

THEME	SUBTHEME	ILLUSTRATIVE QUOTE(S)
*Expanding Perspectives*	*Ethical global health*	“My view is biased – I have spent such a limited amount of time here and I see only the short‑term. The truth is that so much progress has been made, progress that can only be made when people commit to a community for a long period of time, and that is the macroscopic view that people saw and continue to see in these developing communities.” “If we don’t work to let our hearts break for the hurting, then our board meetings about sustainability and bilateral exchange will be less informed, less motivated, and less effective.” “We have discussed how it is easy to judge a care system based on its apparent ‘low‑hanging fruit’ problems, but it is more important to look past these snap judgements and potential ‘quick fixes’ to look into the depths of what is working and what isn’t.”
	*Impactful patient experiences*	“They weren’t tears of sadness but of joy. Joy in a chance at life. Joy in new beginning. She was surely dead within the year if not for an antiretroviral treatment. This big system that has taken years to develop was changing lives like hers.” “From the child with xeroderma pigmentosum to the patients with pneumocystis pneumonia or hydatid cysts, it has not only been a learning experience being on the wards but also a reminder of why many of us chose to go into the medical profession.” “My time spent in the [HIV Resistance] clinic was a rollercoaster of emotions. I did not expect to find myself face‑to‑face with one of [ARVs] failures, especially in the form of a very young child.”
	*Cultural humility*	“I was forced to acknowledge my own privilege, question my own values, and scrutinize how the media chooses to portray systems and cultures foreign to our own. While the Kenyan system is one that is resource‑poor, it is not inferior in knowledge, it is not inferior in joy, it is not inferior in quality of life.” “My time spent in the [HIV Resistance] clinic was a rollercoaster of emotions. I did not expect to find myself face‑to‑face with one of [ARVs] failures, especially in the form of a very young child.”
	*Health equity*	“The trip taught me much about the importance of access to health care for everyone. Seeing patients with cancer so advanced that they could barely walk into the clinics underscores the importance of preventative care and not just acute care.” “Cases like this make it more apparent to me that there is an increasing need for universal access to safe and affordable surgical and anesthetic care to save lives and stimulate growth with communities around the world… a lack of affordable and accessible surgical care prevents people from flourishing and living to their potential.”
*Motivation for service‑oriented career*	*Global health*	“To see how the health and quality of living can be improved from within the hospital to the communities within villages is powerful and a testament to why we should all be interested and strive to become involved in global health.”
	*Optimism*	“Even though there is so much to be done, there is so much hope, and that is what draws people here, and what makes me want to come back.”
	*Underserved*	“The AMPATH mission is something I carry close to my heart – to improve the health of people in underserved communities by leading with care. In the future, I hope to encourage broader application of the AMPATH model and its principles to developing sustainable institutional partnerships.”
*Interpersonal skills*	*Relationship building*	“Above all, it has become abundantly clear to me throughout my short time here that little can be accomplished alone, but what can be accomplished in consortium and partnership with open minds and open hearts seems to be nearly limitless.” “What was so special about this Chama visit was we got to see the community health volunteer run the group as she usually would, in Swahili and the local language. I felt even more welcomed into the group being able to experience how it usually runs and getting to focus more of my attention on the nonverbal ways the women interacted, visibly demonstrating trust and mutual respect.”
	*Collaboration*	“[Those times where] I was privileged to connect and collaborate with my new Kenyan friends were the interactions where I learned the most this summer.”
*Innovation*		“[My Kenyan co‑workers] valued this process because it took them beyond the routine and brought up the ‘why’ behind what they do. I think this is such a good idea for any group, board, school, hospital, nonprofit, etc. Periodically, a staff/team should question why they do the things they do to keep their values and past lessons in focus.”
*Adaptability*		“For example, instead of using a human mannequin with ribs for teaching how to insert a chest tube, I was tasked with finding a suitable replacement that would also be affordable so that future ATLS classes could create them without much difficulty. However, it has been pretty hard trying to explain to the butcher what cut of goat meat or pork meat I wanted.” “While the [healthcare] system is one that is far from what I was accustomed to, despite the initial shock and instinctive comparison to medical care at home, I learned to quickly adjust to how the system works and flows.”

## Discussion

This study highlights the personal and professional value of embedding STEGHs within long‑term partnerships in global health education and the benefit of expanded access to such opportunities for preclinical trainees. Trainees were impacted by both traditional pedagogic educational opportunities such as lectures on topics in global health, and personal transformative experiences including the development of cultural humility and refining goals for the future. These two categories appeared in roughly equivalent proportions of participants’ reflections.

While scholarly work is less commonly described and sometimes explicitly discouraged during clinical rotations, in this preclinical global health experience, most participants formally disseminated their project contributions. While still maintaining high standards for scholarly work conducted by trainees through well‑vetted standard operating procedures that follow best practices for ethical global health research, preclinical students can uniquely integrate into collaborative research early in their careers and may create future GH physician–researchers who continue to champion local counterpart inclusion in research.

Previous studies have shown that STEGHs influence career trajectories and practice settings for clinical medical students and residents [8–10, 13]. One mechanism is transformational learning, as described by Litzelman et al.’s evaluation of clinical students’ reflections after an STEGH, wherein early exposure to STEGHs changes or strengthens the students’ worldview, impacting career decisions. Emergent themes in this study suggest STEGHs influence preclinical medical students’ motivation for service‑oriented careers, including global health. Interestingly, they felt less strongly that the program changed their career trajectory, possibly due to a pre‑existing interest in global health influencing their participation in the SS program. For those who continued careers in global health, the data suggest a moderate to strong impact of the SS program on career decisions. Altogether, the SS program affirms preclinical medical students’ career trajectories through ethical educational experiences and transformative learning.

Following the implementation of binary pass–fail scoring for the United States Medical Licensing Examination (USMLE) step in 2022, it is believed that residency programs may place greater emphasis on factors such as applicants’ familiarity with the program through home or away rotations, research endeavors, and letters of recommendation [28]. Students may feel pressure to solidify their career interests earlier owing to this change. For preclinical medical students interested in global health, this could lead to participation in ethically questionable international experiences. The SS program is one solution to these new demands, offering ethical early exposure to global health.

This single‑center study provides an example of how other centers may develop ethical and impactful global health programs for preclinical medical students. The success of a program hinges on the adherence to existing ethical frameworks for GH, most crucially embedding any student activities within a long‑term, bilateral partnership with a host institution. Consensus‑based best practice guidelines for STEGHS focus on four core ethical principles that the Slemenda Scholars program embodies and that can serve as a foundation for replication and expansion [20, 23, 29]:

- **Skills‑building in cross‑cultural effectiveness and cultural humility:** Slemenda Scholars complete cultural and clinical orientation, are encouraged to undertake language education, and participate in written reflection, as well as faculty‑led critical reflection throughout and after the experience.
- **Bidirectional participatory relationships:** Scholars are placed in Kenyan‑led teams, ensuring counterpart relationships and perspective sharing. Scholars are encouraged to build peer relationships with Kenyan medical students and trainees – this aspect was repeatedly identified as one of the most meaningful aspects by trainees in our study.
- **Local capacity‑building:** Student scholarly activity is done in conjunction with long‑term faculty from both host and sending institutions, and representatives from all institutions are included in all publications. While to date it has not been financially feasible to bring Kenyan preclinical students to the United States for an identical experience, the institutional partnership supports Kenyan medical training in a myriad of ways, including medical school scholarships, stethoscopes donated to each class of medical students, and faculty support. The institutional partnership measures success by the health of the host population and invests in care programs that benefit patients.
- **Long‑term sustainability:** Faculty from the sending institution are stationed at the host site for 1–3 years to offload teaching and supervisory activities associated with visiting trainees. Preclinical students are included in educational activities for clinical students, leading to natural mentorship. Costs for the program are shared between a philanthropic global health foundation and an institutional research program for medical students at the sending institution. A central coordinating body (Indiana University Center for Global Health Equity) provides administrative support for logistics.

This study affirms that a preclinical global health program can lead to countless positive outcomes, including developing interpersonal and professional skills and influencing the selection of careers in global health or community service.

### Limitations of this study

The publicly available nature of online narratives describing the SS program may influence their content, though the content of the public narratives did not differ substantially from the anonymous open‑ended responses in the survey. Several authors’ (MG, DR, and MVM) own narrative reflections were excluded from the analysis, but it is possible that their personal experiences as Slemenda Scholars could have unconsciously influenced emergent themes. Retrospective design includes a risk of recall bias, especially for participants who completed the program years ago. Selection bias may also be present, given that those scholars who had more positive experiences were more likely to voluntarily fill out the survey. Researchers noted verbiage from survey questions appeared in some open‑ended question responses, which suggests possible question‑order bias.

Over the 25+ years of its existence, the SS program has evolved and the exact composition of didactics, experiential opportunities, availability of other learners that might facilitate or hinder group processing, and emphasis on scholarly output have varied over time and for each participant. Thus, data from decades of participants may be heterogeneous, and cumulative analysis may not best characterize the salience of certain themes for certain time periods.

### Future directions

Much has been written about how to create ethical STEGHs, and as demand for these experiences grows among medical students, there is an increasing need for best practices in evaluating the quality of existing programs’ ethics, outputs, and impacts. Moreover, there is an urgent need to creatively consider how to increase the capacity of preclinical global health programs to reach more learners who would benefit; virtual and domestic “local–global” experiences are being explored. While there is a robust educational exchange of residents, faculty, and students in their clinical years between IU, Moi University of Kenya, and other AMPATH consortium schools, opportunities for Kenyan preclinical students to rotate in the United States are currently unavailable. Exploring a reciprocal program for the exchange of preclinical Kenyan medical students to the United States is a goal and priority.

## Conclusions

With time at a premium in medical training, early transformative, immersive learning experiences, such as those provided by the SS program, are essential to students’ personal and professional development, and are an important contribution to developing a healthcare workforce that is prepared to work for health for all.

## Data Availability

The datasets generated and analyzed during this study are available from the corresponding author upon reasonable request.

## Role of the Authors

All authors were involved in the planning and execution of the study and had full access to the data. All authors contributed to the manuscript and its review.
